# Parallel‐Meta Suite: Interactive and rapid microbiome data analysis on multiple platforms

**DOI:** 10.1002/imt2.1

**Published:** 2022-03-06

**Authors:** Yuzhu Chen, Jian Li, Yufeng Zhang, Mingqian Zhang, Zheng Sun, Gongchao Jing, Shi Huang, Xiaoquan Su

**Affiliations:** ^1^ College of Computer Science and Technology Qingdao University Qingdao Shandong China; ^2^ Single‐Cell Center, Qingdao Institute of BioEnergy and Bioprocess Technology Chinese Academy of Sciences Qingdao Shandong China; ^3^ Faculty of Dentistry The University of Hong Kong Hong Kong Hong Kong SAR China

**Keywords:** microbiome, multiplatform, parallel computing, visualization, workflow

## Abstract

Massive microbiome sequencing data has been generated, which elucidates associations between microbes and their environmental phenotypes such as host health or ecosystem status. Outstanding bioinformatic tools are the basis to decipher the biological information hidden under microbiome data. However, most approaches placed difficulties on the accessibility to nonprofessional users. On the other side, the computing throughput has become a significant bottleneck of many analytical pipelines in processing large‐scale datasets. In this study, we introduce Parallel‐Meta Suite (PMS), an interactive software package for fast and comprehensive microbiome data analysis, visualization, and interpretation. It covers a wide array of functions for data preprocessing, statistics, visualization by state‐of‐the‐art algorithms in a user‐friendly graphical interface, which is accessible to diverse users. To meet the rapidly increasing computational demands, the entire procedure of PMS has been optimized by a parallel computing scheme, enabling the rapid processing of thousands of samples. PMS is compatible with multiple platforms, and an installer has been integrated for full‐automatic installation.

## Highlights

- Parallel‐Meta Suite (PMS) is an easy‐to‐use software package for fast and comprehensive microbiome data analysis on multiple platforms.
- PMS covers a wide array of functions for data preprocessing, statistics, visualization by state‐of‐the‐art algorithms.
- The entire procedure of PMS is optimized by a parallel computing scheme that enables the rapid processing of thousands of microbiomes.

## Graphical abstract

Parallel‐Meta Suite (PMS) is an easy‐to‐use software package for fast and comprehensive microbiome data analysis on multiple platforms. It covers a wide array of functions for data pre‐processing, statistics, visualization by state‐of‐the‐art algorithms. The entire procedure of PMS is optimized by a parallel computing scheme that enables the rapid processing of thousands of microbiomes.

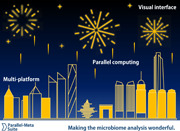

## INTRODUCTION

Excellent bioinformatics tools are essential to deciphering the biological pattern hidden under microbiome big‐data, by which we can interpret the associations between microbial communities and their surroundings like environmental conditions or human health status [1]. In the past decade, functions of bioinformatical tools for microbiome have been largely expanded from basic taxonomy annotation to downstream diversity analysis and biomarker selection, enabling microbiome data mining for broad purposes. Nevertheless, complicated command‐based operations of such highly multifunctional toolkits like QIIME [2] or old version Parallel‐Meta [3] place barriers for the nonspecialist to manipulate, or even get started. On the other side, the sequencing cost has been substantially reduced over the past years. It promotes the surveys of microbes from various habitats or large cohorts like the Earth Microbiome Project [4] or American Gut Project [5], while also increasing the requirement of computational throughput and efficiency for data processing.

In this situation, some approaches, for example, q2studio [2] provides a graph‐based user interface (GUI) to improve usability. However, such a graphical interface always relies on many dependencies and specific operating system environments during both installation and running, which may not be supported in some cases like remote‐login servers for big‐data handling. An alternative solution is online web services with GUI such as Galaxy [6] or gcMeta [7]. Notably, inevitable network latency of data transmission and shared computing resources limit the data size, especially for a large volume of microbiome sequencing data. In addition, data privacy and security issues are also concerned when using open online platforms with unpublished datasets.

To tackle these challenges, here we propose Parallel‐Meta Suite (PMS), an interactive software package for rapid and comprehensive microbiome analysis. PMS has been significantly enhanced and re‐engineered based on the well‐established marker‐gene‐based analysis protocols and workflows [3, 8], featuring the improved accessibility to a variety of users with a user‐friendly graphical interface, and the optimized analysis performance by a parallel computing scheme that has been tested in many application scenarios. In addition, to solve the installation issues that many bioinformatic tools suffered from, such as package dependency, system setup, and source code compiling, we also developed an automatic installer that helps users can easily configure and install PMS. Now the latest version of PMS software is released at GitHub (https://github.com/qdu-bioinfo/parallel-meta-suite) and Gitee (https://gitee.com/qdu-bioinfo/parallel-meta-suite), and a demo data set is also available in the package for trial use.

## METHODS

**Figure 1:**
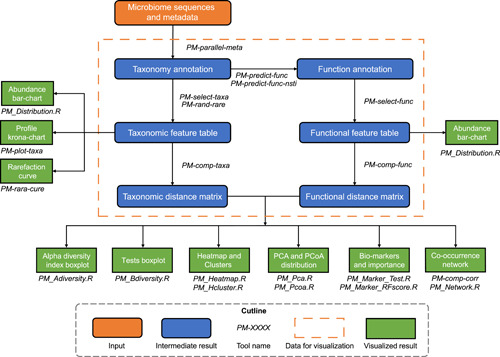
Workflow of PMS for whole pipeline and visualization. Details of tools are introduced in Table S1. PMS, Parallel‐Meta Suite

The analytical workflow of PMS is illustrated in Figure 1. PMS can take metagenomic shotgun or amplicon sequences as the original input. For shotgun sequences, marker gene fragments (e.g., 16S or 18S ribosomal RNA [rRNA] gene) are identified and extracted by Hidden Markov Models [9]. For amplicon sequences, PMS performs amplicon sequence variants (ASVs) denoizing [10] and de‐chimera [11] for marker genes to avoid sequencing inaccuracy (this step is optional for shotgun sequences but the default setup is disabled). Then sequences are aligned against reference databases by the built‐in vsearch [12] for profiling and taxonomy annotating from kingdom level to species level. The relative abundance of community members on each taxonomy level is also corrected using marker gene copy number normalization. The gene families are inferred into KEGG Orthology using PICRUSt algorithm [13], and metabolic pathways are annotated by KEGG BRITE hierarchy. PMS also measures the prediction accuracy of functions by the Nearest Sequenced Taxonomy Index value [14], which is calculated by the sum of distances between operational taxonomic units and their nearest individually sequenced relatives in the phylogenetic architecture.

The comprehensive taxonomy is visualized by Krona [15] and bar charts. Then microbial diversity analysis, biomarker selection, and co‐occurrence network construction are performed on specific taxonomy or pathway levels chosen by users. For alpha diversity, Shannon, Simpson, and Chao1 indexes of each sample are calculated. Alpha indexes are illustrated into boxplots with Wilcoxon or Kruskal rank‐sum test for discrete metadata (e.g., type, status, gender, etc.), or curve plots for numerical variables (e.g., age, body mass index, PH, etc.) with regression analysis. For beta diversity, pairwise distance matrices are calculated by weighted/unweighted Meta‐Storms [16] algorithm (for taxonomy) or Hierarchical Meta‐Storms [17] (for function) and plotted by heatmap. After that, the beta‐diversity pattern is demonstrated by principal co‐ordinate analysis and principal component analysis diagram and measured by PERMANOVA and ANOSIM tests for discrete metadata, or regression analysis on numerical metadata variables and distance values. For biomarker analysis, PMS uses Wilcoxon or Kruskal rank‐sum test to select out organisms or gene units with significant differences among different groups (discrete metadata) as candidates, which are then ranked by Random Forest [18, 19] importance. Microbiome features that are strongly correlated with numerical metadata variables are also selected out as biomarkers by regression analysis. In co‐occurrence networks, nodes are community features (e.g., a taxon), and edges represent their Spearman correlation. Then the network density, diameter, radius, and centralization are computed to quantify the network property.

## RESULTS

### Key features of PMS

**Figure 2:**
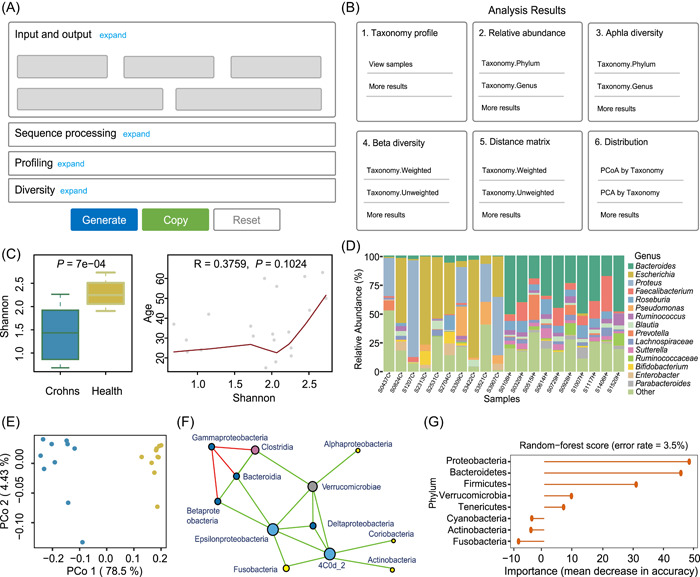
GUI‐based visualization of PMS. (A) The interactive configuration guides. (B) The result viewer. (C) Alpha diversity calculation and its association with key phenotypes. (D) Sample‐level relative abundance profiles. (E) Principal co‐ordinate analysis shows the unsupervised sample clustering in reduced dimensions (beta‐diversity analysis). (F) Co‐occurrence network analysis. (G) Biomarkers selection by internal importance scores produced by a supervised machine‐learning algorithm of Random Forest. GUI, graph‐based user interface; PMS, Parallel‐Meta Suite

PMS provides a user‐friendly GUI (Figure 2) for data analysis configuration and detailed results interpretation. This GUI enables users to easily get started with an example data set, and further simplifies the learning curve for advanced usages with customized parameters. Using web‐page‐based visualization, PMS is compatible with different environments (e.g., local system or remote login server) and multiple systems (e.g., Linux, Mac, or Windows). As a highly integrated and automatic workflow (refer to Methods section for details), PMS implements a variety of state‐of‐the‐art algorithms and analysis strategies in the microbiome study, including advanced sequence processing (e.g., metagenome marker‐gene extraction, analyzing denoized ASVs, prediction of functional profiles from 16S data [13], alpha and beta diversity calculation and multivariate statistical analysis [17, 20], biomarker selection, and evaluation, and co‐occurrence network analysis, and so forth. The marker‐gene references are also updated and expanded by databases of GreenGenes [21], SILVA [22], Oral‐core [23], SILVA‐18S [22], and ITS [24] that contain full‐length 16S rRNA, 18S rRNA, and ITS sequences. Moreover, PMS is fully parallelized and optimized, where the whole processing pipeline of 14,000 16S samples could be accomplished in 43 h on a single computing node (refer to Parallel Computing and Speed section for details).

### Implementation of GUI and parallel computing

The GUI of PMS software consists of two components, an interactive configuration guide (Figure 2A), and a visualized result viewer (Figure 2B). The configuration guide is integrated in the software package. As a user‐friendly graphical interface, it sorts all analytical parameters into a well‐organized structure according to the pipeline. Initially, all parameters are set with default values, and only basic arguments should be specified (e.g., input/output type and path) for easy startup. Advanced options can be expanded for further adjustment of profiling, diversity analysis, and statistics. Based on users' setup, this configuration guide can generate the corresponding executable command. The result viewer is automatically created in the output directory after the whole pipeline is completed. It displays the categorized results, and visualizes each of them by plots with elaborately designed schemes and colors (Figure 2C–G), providing a direct and clear interpretation of microbiome patterns. This GUI would be highly helpful to nonprofessional users who are not familiar with the command‐line interface or complicated parameters, also provide a better and clearer understanding of the workflow and results. In addition, as the configuration guide and result viewer are accessible via any web browser conveniently, the PMS GUI is highly compatible with multiple platforms including Linux, Mac, and Windows.

The PMS framework is primarily developed by C++ that exhibits superior performance in running efficiency and memory usage than script‐based programming languages. Taking the parameters parsed from the GUI, this framework invokes and manages the analytical steps in the workflow. Overall, we optimized this workflow with the parallel computing scheme in two different ways. (1) The computing steps (Table S1A) related to taxonomic identification and abundance estimation, function prediction, and distance matrix calculation that were implemented by C/C++ have been directly parallelized by the C‐based OpenMP library. (2) We also parallelized the statistical steps (Table S1B) related to alpha and beta diversity calculation and statistical tests, biomarker selection, and plotting that were developed by CRAN‐R (https://www.R-project.org). Each of the R scripts implementing any of these analyses is assigned to a thread by this framework, then all threads can be launched simultaneously. The number of threads is automatically set as the CPU‐core number for full utilization of hardware resources, while can also be manually controlled by users.

### Usages in different scenarios

**Figure 3:**
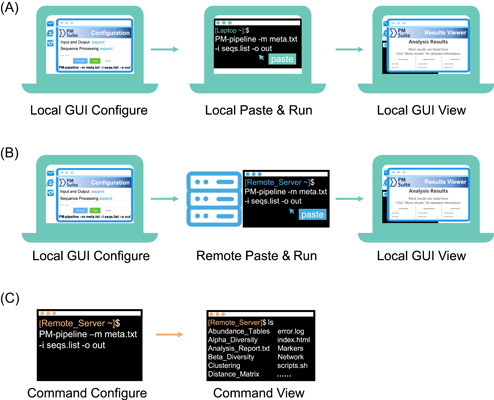
Three typical usage modes of PMS in different scenarios and platforms. (A) Local GUI configures and run. (B) Local GUI configures and remote run. (C) Command‐line configuration and run (for either locally or remotely). GUI, graph‐based user interface; PMS, Parallel‐Meta Suite

Here, we demonstrate the usages and experience of PMS in three typical scenarios (Figure 3) under different computing platforms and environments.

#### Scenario I: GUI‐based configuration and run in a local computer

PMS can be installed and performed in a “local” personal computer (e.g., a laptop) to process a small number of samples (e.g., less than 200). Local GUI‐based usage (Figure 3A) is applicable to operating systems of Linux (GUI desktop installed), Mac, or Windows 10+(Subsystem for Linux [WSL] installed). The configuration guide can be accessed via the “index.html” page in the Homepage folder of the software. Users can either keep the default options or adjust parameters according to actual requirements. After configuration, by clicking the “Generate” and the “Copy” button at the bottom of the page, a valid command is generated and copied in the clipboard. Then this single‐line command can be pasted in the local terminal to successfully run the PMS pipeline without other operations. In the output directory, the visualized result viewer is also named as “index.html,” as well as all raw results (e.g., relative abundance table, distance matrix, etc.) that are by default kept for further in‐depth data mining or meta‐analysis. In addition, the analysis summary, work log, and detailed step‐by‐step workflow script are also provided in the result folder.

#### Scenario II: Local GUI‐based configuration and remote run in a server

Since processing many samples (e.g., >1000) is time and resource‐consuming, we recommend running the PMS pipeline in more powerful servers. Usually, such servers need a remote login (e.g., via SSH) and only provide a command‐based terminal to operate the pipeline. In this case (Figure 3B), users shall appropriately install PMS in the server, download and open the GUI configuration guide (“index.html” in the software package) in the local computer to generate the command and run the commands in the terminal of a remote server. The results can also be transferred to the local computer for browsing like *Scenario I*. Therefore, the pipeline can be easily configurated and performed without massive data transfer.

#### Scenario III: Command‐based configuration and run

PMS also supports command‐line‐based operations in the non‐GUI conditions, and typically for experienced users (Figure 3C). To meet the increasingly user‐specific requirements in the metagenome analysis, this pipeline can work in highly flexible settings, for example, running each step with customized parameters, or performing selected steps of the workflow. This is available via command‐based terminals either locally or remotely. The command‐line interface also provides tutorials that describe the detailed usage and the brief help information for the pipeline in every single step (e.g., parsing the “‐h” argument for each program in Table S1).

### Case studies and results

We employed two example datasets to demonstrate the capability of PMS in decoding the microbiome profiles and associating ecological patterns with key metadata. Both datasets are collected from previously published works, such that the accuracy and reliability of analysis results with PMS can be verified.

#### Case I: Variation of indoor microbiome before and after hospital opening

**Figure 4:**
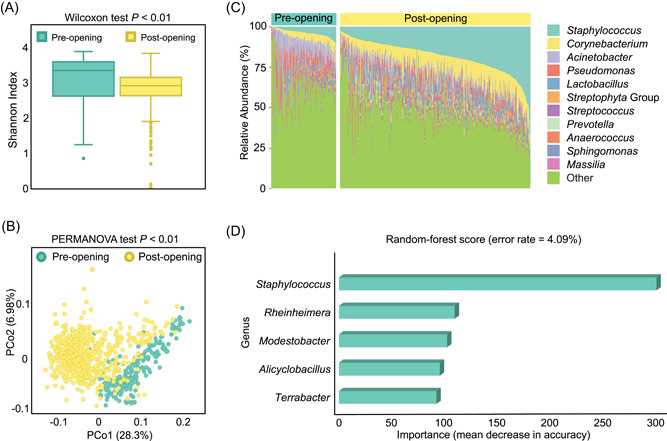
Variation of indoor microbiome before and after hospital opening. (A) Shannon index of alpha diversity decreased after hospital opening. Wilcoxon test *p* < 0.01 (*p* < 0.05 denotes significant difference). (B) Overall beta diversity significantly different between preopening and post opening states based on weighted Meta‐Storms distance. PERMANOVA test *p* < 0.01 (*p* < 0.05 denotes significant difference). (C) Dynamics of relative abundances on genus level between two time points. (D) Five bacterial genera were selected as biomarkers that can distinguish two time points. The *x*‐axis is the importance score (mean decrease in accuracy) produced by the Random Forest model that evaluates the importance of each biomarker on distinguishing different hospital status

Data set 1 contains 894 16S‐amplicon microbiome samples from a hospital's indoor environment before and after opening (Table 1). We performed a PMS pipeline with all default parameters (refer to Table S2 for details). From the results, we can observe that the Shannon alpha diversity decreased after the hospital was opened (Figure 4A; Wilcoxon test *p* < 0.01), and the overall community significantly shifted in the beta diversity (Figure 4B; weighted Meta‐Storms distance, PERMANOVA test *p* < 0.01), which have been reported by Lax et al. [25] The predictive functional diversity also followed the similar trend as the taxonomy (Figure S1). Such microbial dynamics between the two time points can also be illustrated by the variation of relative abundance (Figure 4C). Using statistical tests and a machine learning pipeline, PMS also identified the most important microbes that contributed to distinguishing such ecological changes in the hospital surface from the preopening to post opening state, for example, *Staphylococcus*, *Rheinheimera*, and *Modestobacter*. This machine‐learning model achieved an accuracy of 95.91% (error rate = 4.09%) in differentiating the status of indoor samples (Figure 4D) on the genus level.

**Table 1:** Detailed information of the test datasets

Data set	Study	No. of samples	No. of sequences	Platform
Data set 1	Lax et al., *Sci. Trans. Med.*, 2017 [25]	894	39,192,961	Illumina
Data set 2	Hacquard et al., *Cell Host & Microbe*, 2015 [26]	2556	223,845,875	Illumina and Roche 454
Data set 3	American Gut Project [5]	14,000	478,917,759	Illumina

#### Case 2: Meta‐analysis of the microbiome from multiple habitats

**Figure 5:**
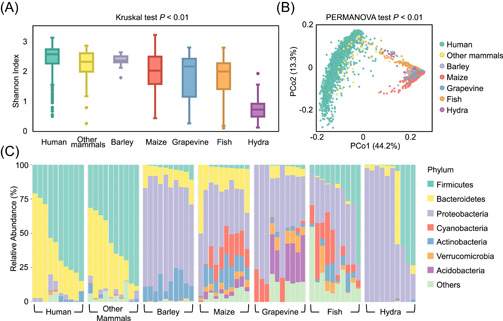
Meta‐analysis of the microbiome from multiple habitats. (A) Alpha diversity among host types was distinct on the Shannon index. Kruskal test *p* < 0.01 (*p* < 0.05 denotes a significant difference). (B) Samples were grouped by habitat in weighted Meta‐Storms distance‐based PCoA pattern. PERMANOVA test *p* < 0.01 (*p* < 0.05 denotes a significant difference). (C) Abundant community members varied among different habitat types. PCoA, principal co‐ordinate analysis

Data set 2 consisted of 2556 host‐associated microbiomes (Table 1) sampled from diverse host species and studies [27, 28, 29, 30, 31, 32, 33, 34], from which we performed a meta‐analysis to systematically investigate the microbial distribution across environmental habitats. AS 16S rDNA sequences were produced by different platforms (i.e., Illumina and Roche 454), ASV denoising and de‐chimera were disabled but other options were kept as default values (refer to Table S2 for details). Results in Figure 5A,B showed that PMS reveals the distinct alpha and beta diversity of microbiomes between host sources or habitat types. This was mainly due to a few overlaps of abundant taxa between mammalian gut and plant roots, whereas fish gut and plant root communities had common microbial members, for example, dominant phyla of *Proteobacteria*, *Cyanobacteria*, and *Actinobacteria* (Figure 5C). Such pattern exhibited a high consistency by previous meta‐analysis studies in Hacquard, et al., *Cell Host & Microbe* 2015 [26]. It is also interesting that the functional alpha and beta diversity produced similar results as taxonomy (Figure S2A,B), however, all samples shared some metabolic pathways at KEGG BRITE Level 2 (Figure S2C), such as Protein families genetic information processing, signaling and cellular processing, carbohydrate metabolism, amino acid metabolism, and energy metabolism.

### Parallel computing and speed

We furtherly evaluated the performance of PMS in parallel computing speed and efficiency using three datasets (Table 1). For Data sets 1 and 2, we set different numbers of CPU threads (1, 10, 20, 40, and 80), respectively, repeated the whole workflow and compared the running time to test the parallel computing efficiency. Data sets 1 and 3 were sequenced by the Illumina platform that were applicable for ASV‐based profiling. Data set 2 contains sequences by both Illumina and Roche 454, so ASV was set as off. Other parameters were kept as the default configuration (Table S2). All speed tests were performed on a single‐node rack server that supports 80 threads (40 physical CPU cores).

**Figure 6:**
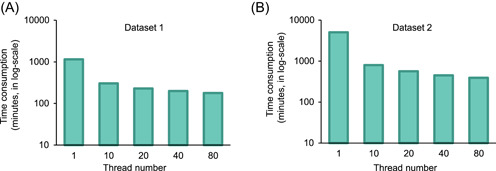
The running time consumption of Parallel‐Meta Suite on different scale datasets

Optimized by dynamic thread scheduling and load balancing for parallel computing, PMS is capable for handling thousands of microbiomes, for example, the entire workflow of Data set 2 with more than 2500 samples can be accomplished in 392 min, and even 14,000 samples of Data set 3 in 43 h. From the results in Figure 6, we observed that the reduction in the run time was linearly associated with the thread numbers, suggesting the high computational efficiency with the parallelization and subtask scheduling strategy. Furthermore, the acceleration ratio was irrelevant to the source or the sequence type of the input samples. Such acceleration demonstrated that PMS can perform taxonomic and functional profiling of input samples in a rapid and timely manner, which is essential to the in‐depth data mining with over 10,000 of samples from different technical backgrounds.

## DISCUSSION

As the microbiome data processing criteria have been well‐updated and ‐established in the past few years, the key focus of bioinformatics tools is shifting from the expansion of functionality to the promotion of usability. As a continuously maintained and iterated software work, PMS aims to provide a delightful working experience for users at all levels, a comprehensive set of microbiome analytical solutions by up‐to‐date approaches or techniques, as well as accelerated performance in handling the large‐scale data. Such advanced features also enable the fast and comprehensive microbiome data analysis from multiple studies, thus contributing to forming the integrated microbiome knowledge base using a wide range of datasets for interdisciplinary cooperation.

In addition, PMS also facilitates in‐depth data mining by its high compatibility to downstream analysis with other state‐of‐the‐arts. First, its data visualization results can offer a clear understanding of microbial diversity patterns associated with key phenotypes and generate certain key hypotheses for downstream analysis or a larger‐scale study. On the other hand, all background raw data is stored in standard or commonly‐used formats for downstream processes for big data mining. For example, the relative abundance tables with different sets of microbial features (e.g., taxonomy or functional pathway) also fit with other microbiome analytical tools or machine learning tools. Such microbiome profiling output can be directly and seamlessly taken by our previously developed tools like Microbiome Search Engine [35] or Meta‐Apo [36], which greatly promotes the data‐driven science [37] in this field.

## CONFLICT OF INTERESTS

The authors declare that there are no conflict of interests.

## AUTHOR CONTRIBUTIONS

Xiaoquan Su conceived the idea. Yuzhu Chen, Jian Li, and Zheng Sun developed the software and algorithm. Yuzhu Chen performed the analysis. Yufeng Zhang, Mingqian Zhang, Zheng Sun, and Gongchao Jing contributed to data collection and curation. Xiaoquan Su, Yuzhu Chen, and S.H. Shi Huang wrote the manuscript.

## Supporting information

Supporting information.

Supporting information.

Supporting information.

Supportiknformation.

## Data Availability

The software package is released at GitHub (https://github.com/qdu-bioinfo/parallel-meta-suite) and Gitee (https://gitee.com/qdu-bioinfo/parallel-meta-suite), in which an installer has been integrated for full‐automatic installation. All datasets used in this manuscript have also been uploaded to online repositories. In each data set package, the “folder” contains the demultiplexed FASTA‐format sequence file of each sample, and their paths are in the list file named “seqs.list.” The “meta.txt” contains the metadata of each sample. Supporting Information (text, figure, table, Chinese translated version, or video) are available online.
