# Supplementary figures and images for: Parallel‐Meta Suite: Interactive and rapid microbiome data analysis on multiple platforms
Source: Imeta. 2022 Mar 6;1(1):e1. doi: 10.1002/imt2.1 (PMC10989749; doi:10.1002/imt2.1)

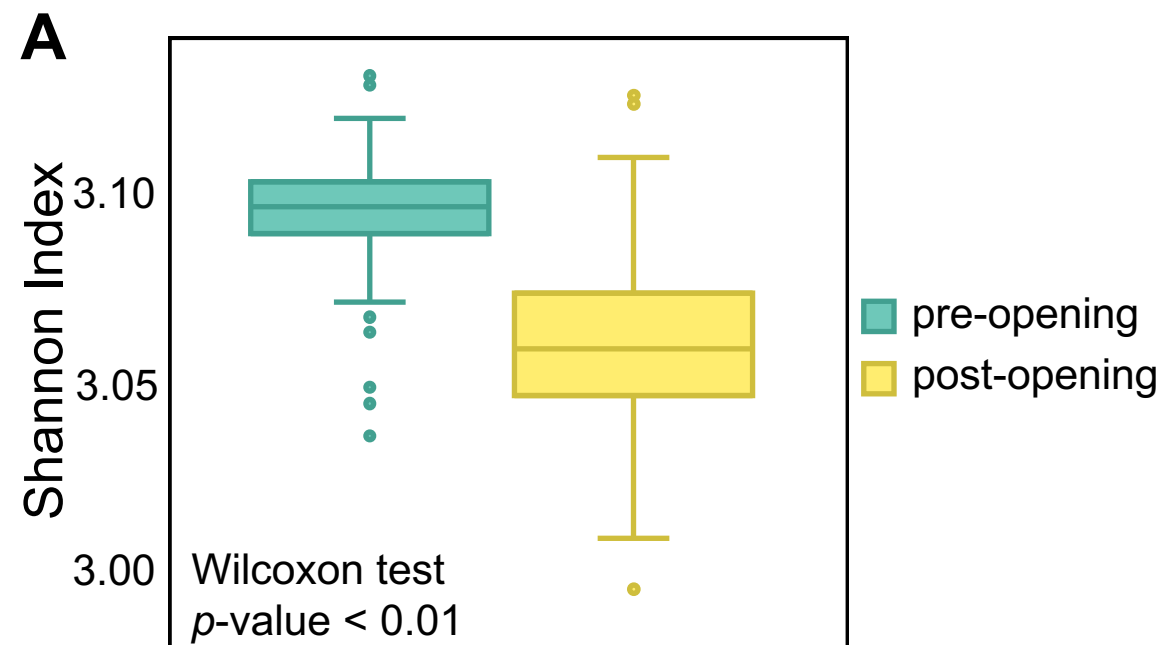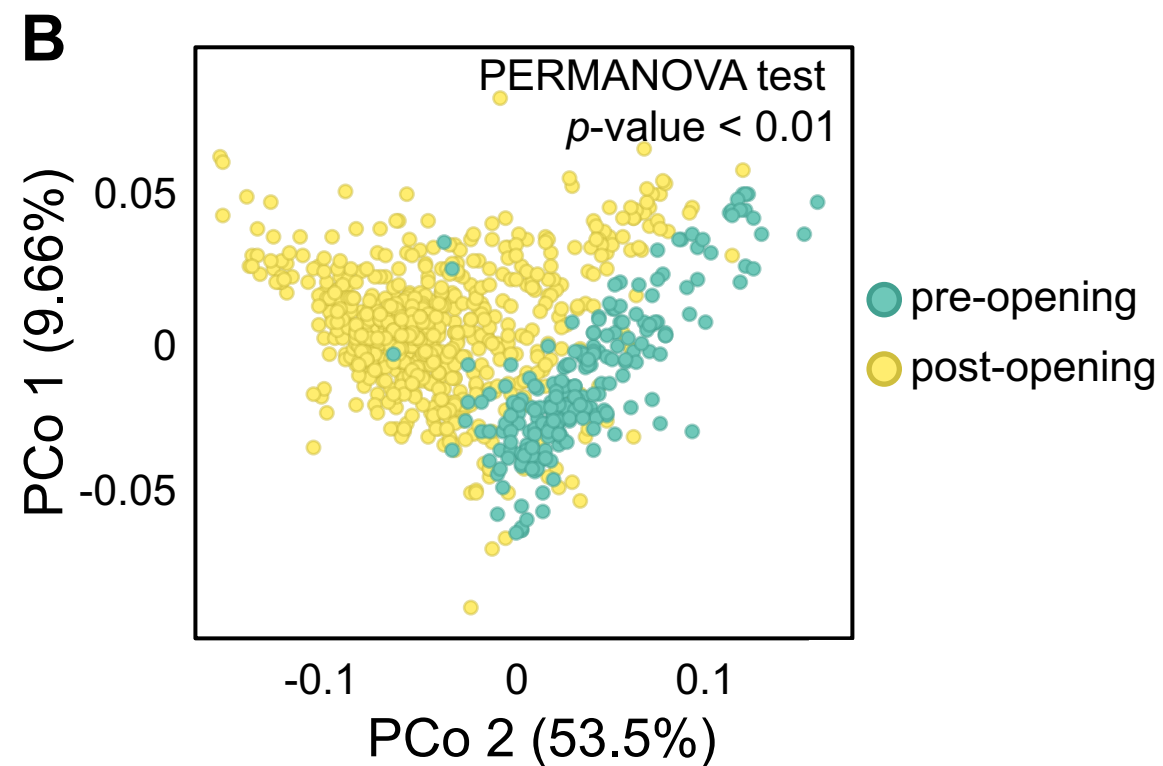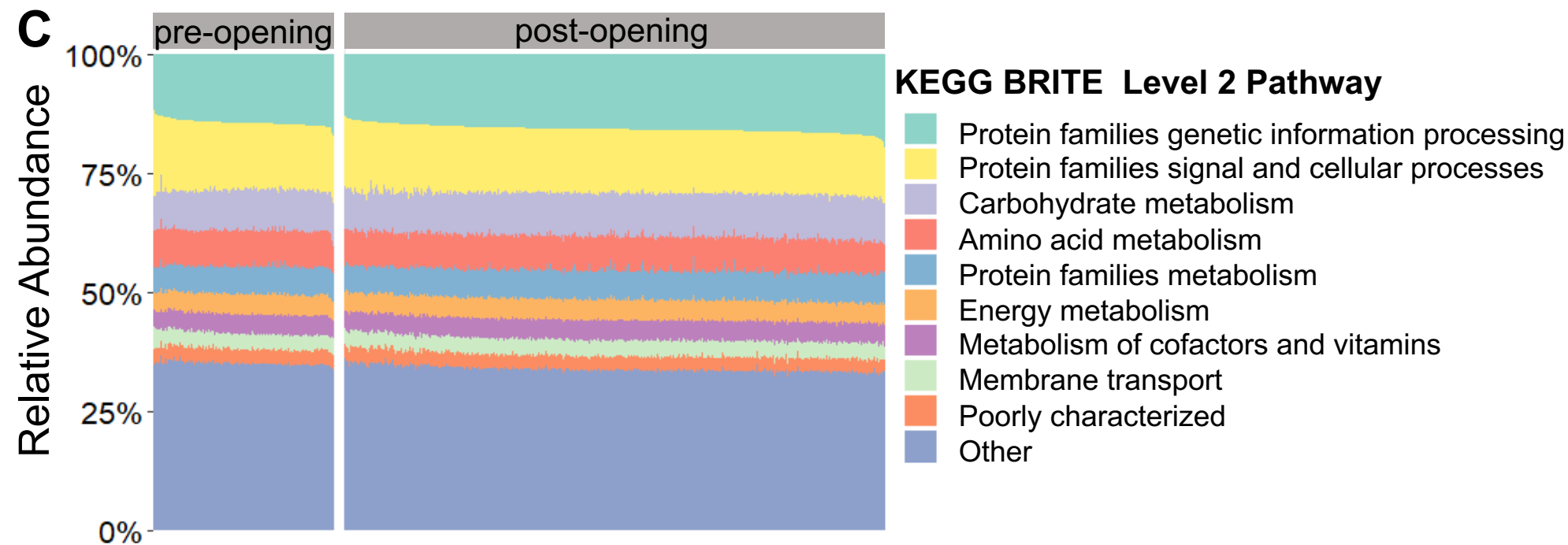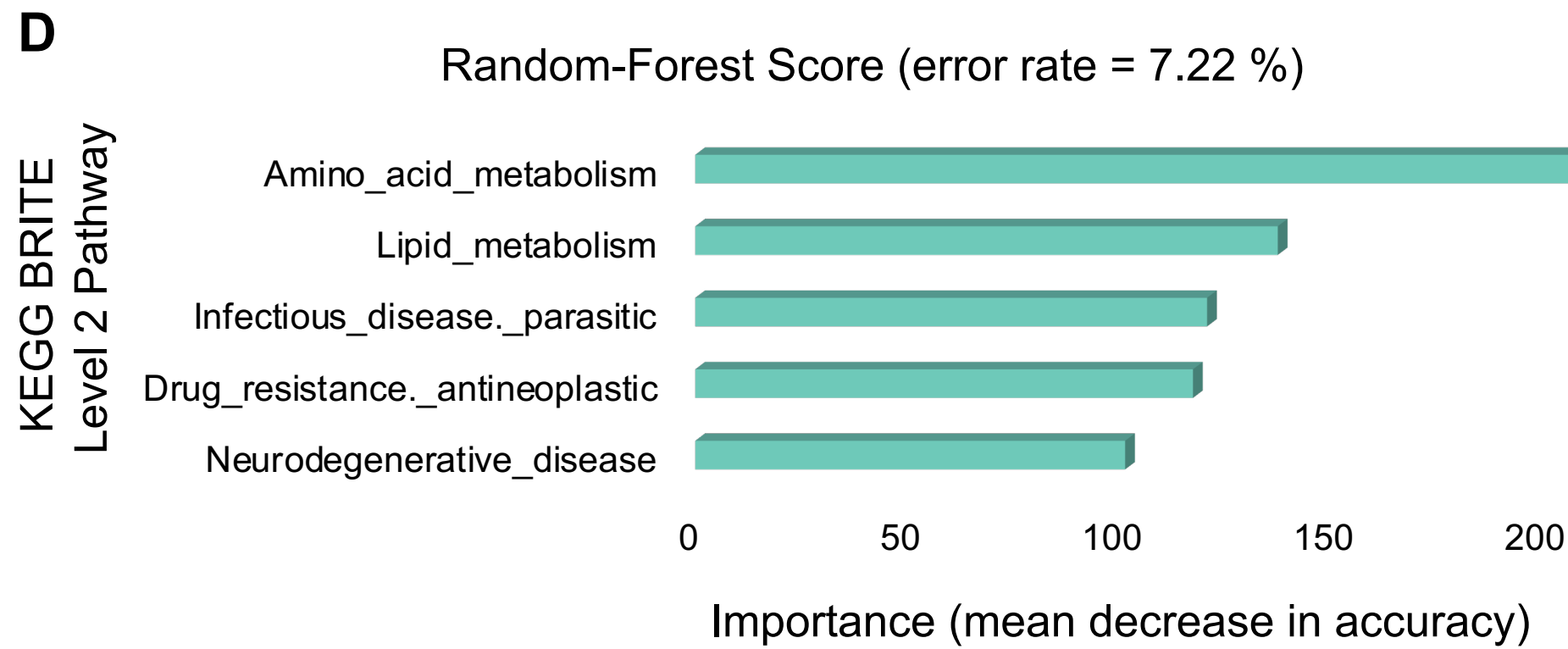

Supplement: Supplementary file 1 — Supporting information. [file IMT2-1-e1-s002.pdf]

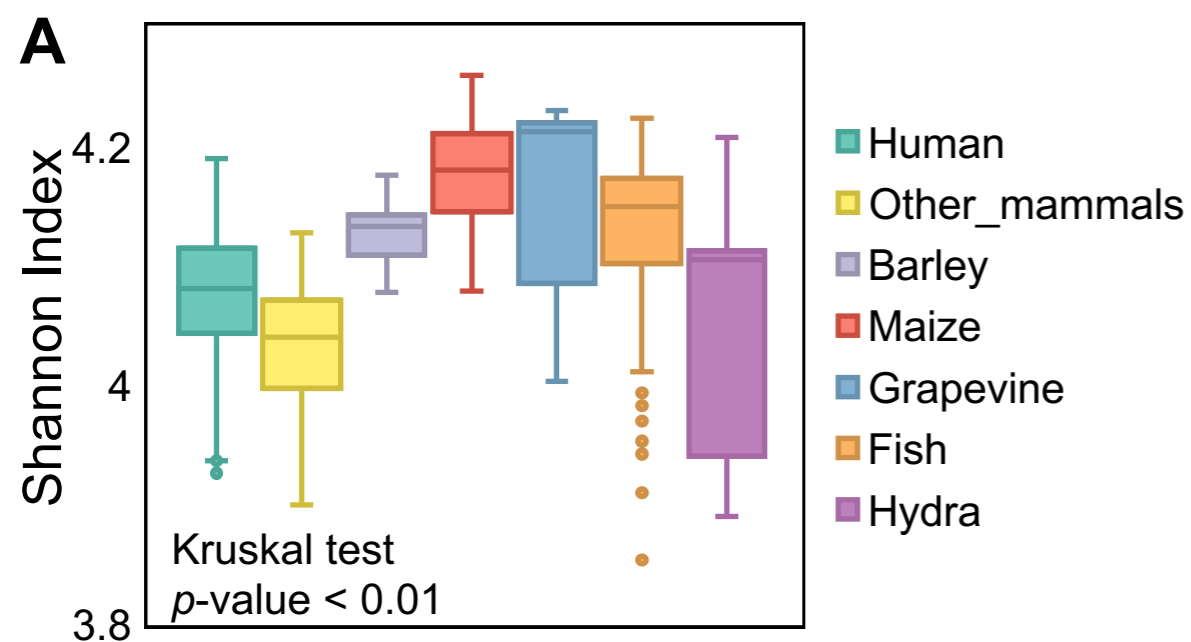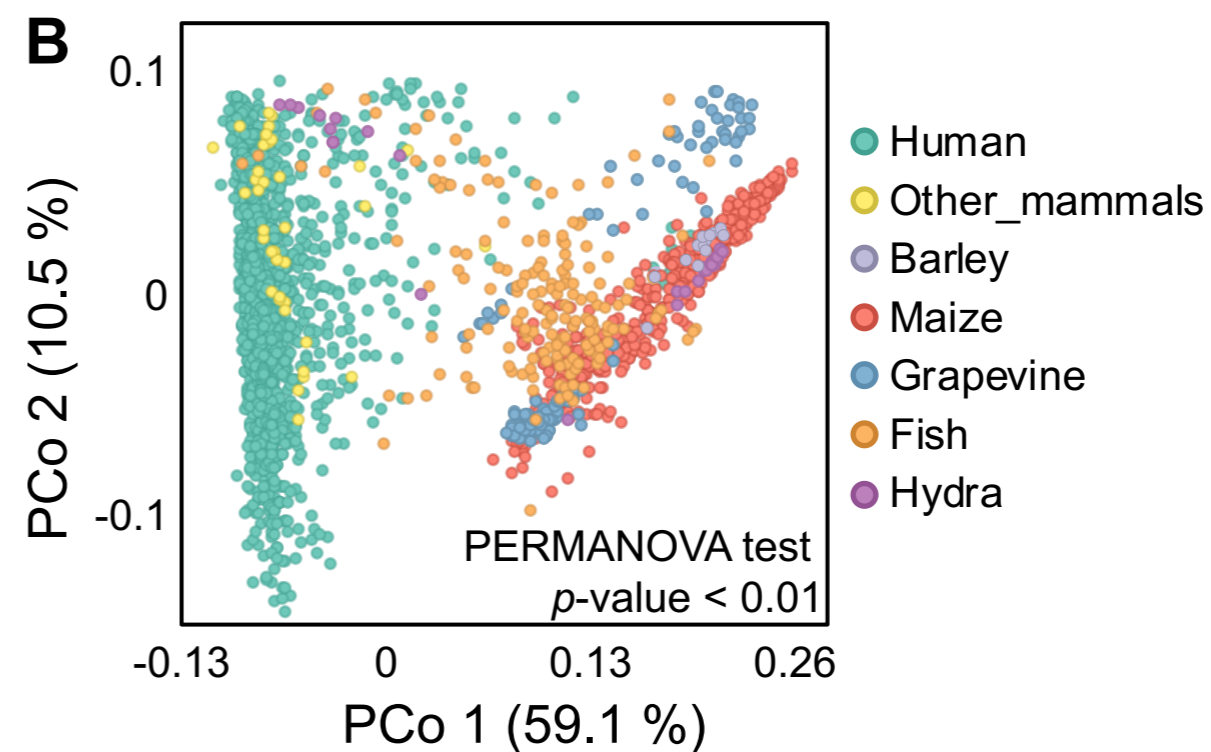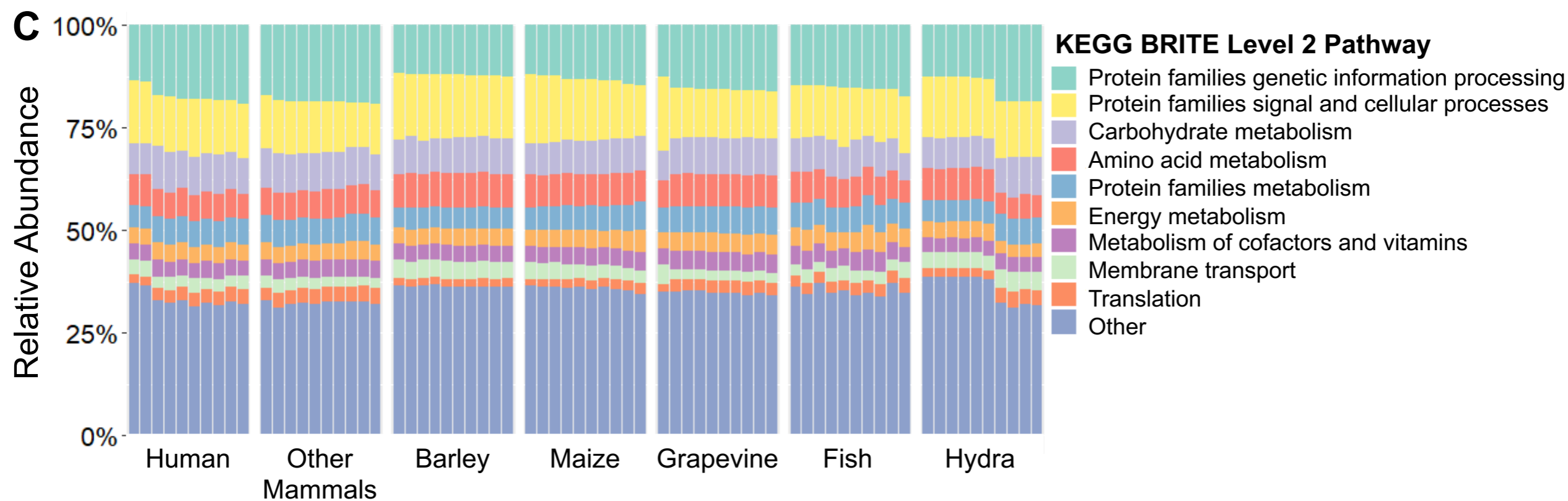

Supplement: Supplementary file 2 — Supporting information. [file IMT2-1-e1-s003.pdf]
