# Supplementary material for: Parallel‐Meta Suite: Interactive and rapid microbiome data analysis on multiple platforms
Source: Imeta. 2022 Mar 6;1(1):e1. doi: 10.1002/imt2.1 (PMC10989749; doi:10.1002/imt2.1)
Supplement: Supplementary file 3 — Supporting information. [file IMT2-1-e1-s001.docx]

**Supplemental Figures**

**
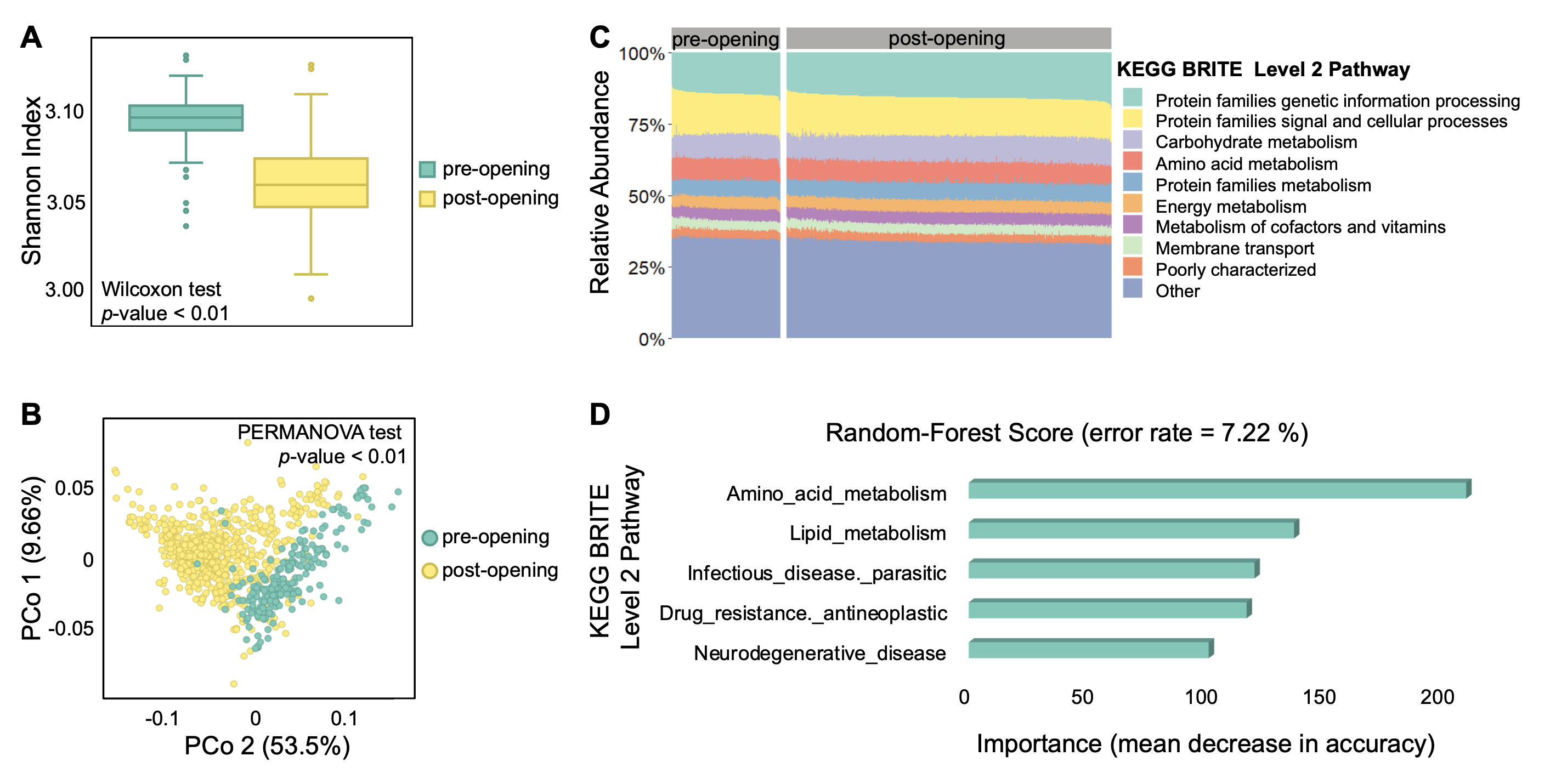
**

**Figure S1. Variation of indoor microbiome before and after hospital opening in function profiles. A**. Shannon index of alpha diversity also decreased after hospital opening. Wilcoxon test *p*-value < 0.01 (*p*-value < 0.05 denotes significant difference). **B**. Overall beta diversity significantly changed based on Hierarchical Meta-Storms distance. PERMANOVA test *p*-value < 0.01 (*p*-value < 0.05 denotes significant difference). **C**. Relative abundances on KEGG BRITE level 2 pathway among two time points. **D**. 5 level-2 pathways were selected as biomarkers to distinguish the hospital status. The x-axis is the importance (mean decrease in accuracy) value from Random Forest model.


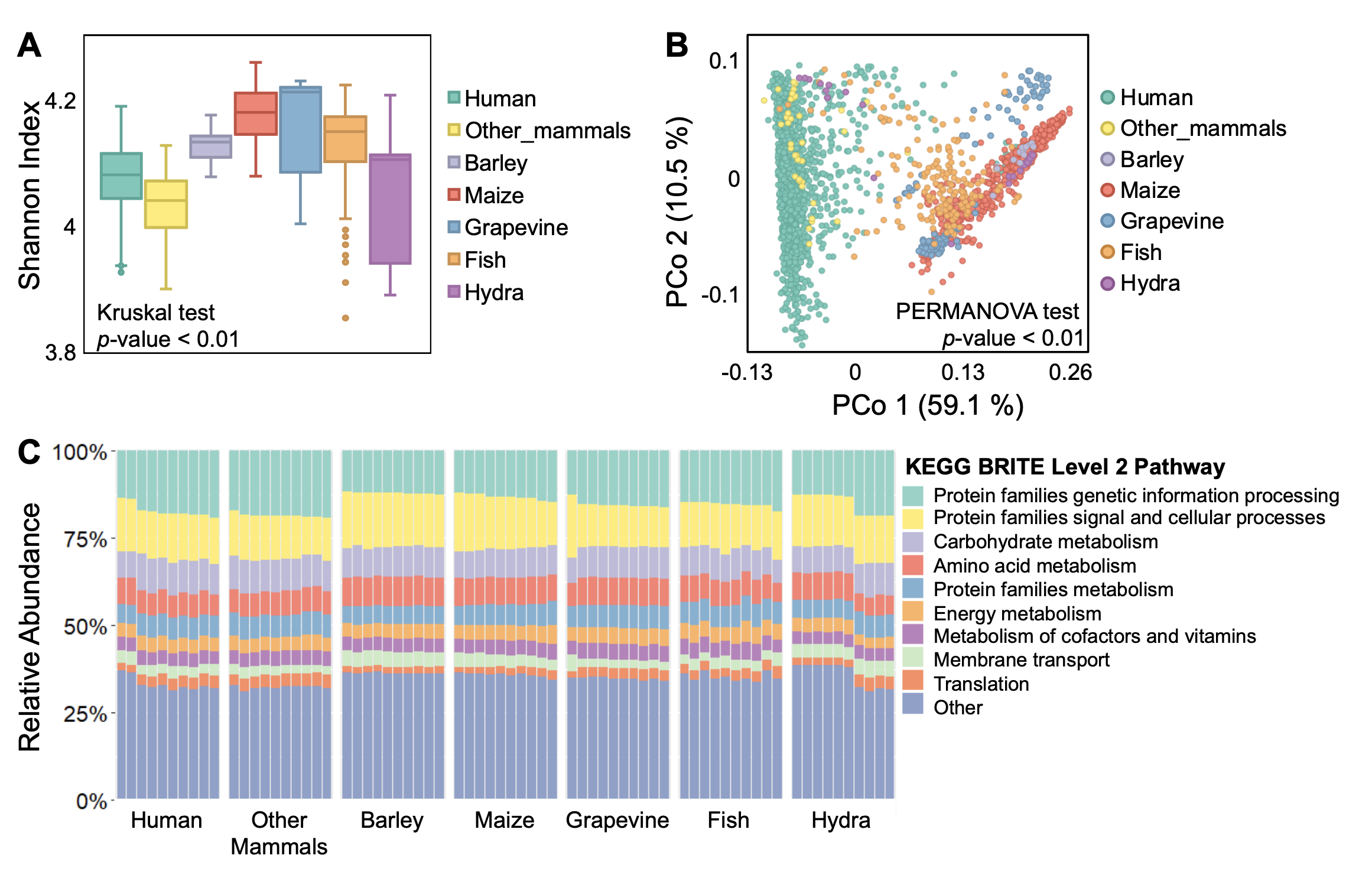


**Figure S2. Meta-analysis of microbiome from multiple habitats in function profiles.** **A**. Alpha diversity among host types were distinct on Shannon index. Kruskal test *p*-value < 0.01 (*p*-value < 0.05 denotes significant difference). **B**. Samples were grouped by habitat in Hierarchical Meta-Storms distance based PCoA pattern. PERMANOVA test *p*-value < 0.01 (*p*-value < 0.05 denotes significant difference). **C**. A few abundant community members varied among different habitat types.
